# Native myocardial T1 mapping using rapid 1.5T T1FLASH and T1 MOLLI during breath-hold and free-breathing

**DOI:** 10.1186/s12872-026-06687-8

**Published:** 2026-09-23

**Authors:** Lena Maria Röwer, Yara Hussen-Demir, Pablo Emilio Verde, Dirk Voit, Jens Frahm, Gerald Antoch, Dirk Klee, Frank Pillekamp

**Affiliations:** 1 Department of Diagnostic and Interventional Radiology, Medical Faculty and University Hospital Düsseldorf, Heinrich Heine University Düsseldorf, Moorenstr. 5, Düsseldorf, 40225 Germany; 2 Coordination Centre for Clinical Trials, University Hospital Düsseldorf, Heinrich Heine University Düsseldorf, Düsseldorf, Germany; 3 Max Planck Institute for Multidisciplinary Sciences, Göttingen, Germany; 4 Cardiovascular Research Institute Düsseldorf, CARID, University Hospital Düsseldorf, Heinrich Heine University, Düsseldorf, Germany

**Keywords:** Cardiac magnetic resonance imaging, Cardiovascular MRI, T1 mapping, Tissue characterization, Free-breathing

## Abstract

**Background:**

Quantitative myocardial T1 mapping is an established technique in cardiovascular magnetic resonance (CMR). However, conventional T1 mapping requires relatively long breath-holds (BH), which may lead to breathing artifacts and reduced image quality in patients with limited BH capacity. The T1 fast low angle shot (T1FLASH) sequence enables myocardial T1 mapping with shorter BH durations.

**Methods:**

In this prospective study, 14 healthy adult volunteers (8 female; mean age 27 ± 4 years) underwent myocardial T1 mapping at 1.5 T using modified Look-Locker inversion recovery with motion correction (T1 MOLLI MOCO) and rapid T1FLASH. Both sequences were acquired during BH and free breathing (FB). Global and segmental myocardial T1 values and image quality were assessed. Interrater, intrarater, and interstudy reproducibility were evaluated. Comfort was assessed using a questionnaire. Statistical analysis included the Shapiro-Wilk test, paired t-test, Pearson correlation, Bland-Altman analysis, intraclass correlation coefficient, Wilcoxon signed-rank test, and Spearman rank correlation (*p* < 0.05).

**Results:**

BH duration was significantly shorter with rapid T1FLASH compared with T1 MOLLI MOCO (3–4 s vs. 12–14 s), and participant comfort was higher with rapid T1FLASH (*p* < 0.05). During BH, global myocardial T1 values were significantly higher with rapid T1FLASH (1030 ± 35 ms) than with T1 MOLLI MOCO (1000 ± 26 ms; *p* < 0.01). The slightly increased variability observed with rapid T1FLASH remained below diagnostically relevant thresholds (4% error margin). Both techniques showed moderate-to-good correlation for global T1 values (*r* = 0.63; *p* < 0.01). Rapid T1FLASH demonstrated good image quality that was comparable for clinical interpretation during BH, although T1 MOLLI MOCO received slightly higher qualitative image scores. During FB, variability of global and segmental T1 measurements increased significantly, with apical segments being more and AHA segments 2 (basal anteroseptal), 5 (basal inferolateral), 8 (mid anteroseptal), 11 (mid inferolateral) less affected by respiratory artifacts. Interrater, intrarater, and interstudy reproducibility were good to excellent.

**Conclusions:**

Rapid T1FLASH demonstrated good agreement with conventional T1 MOLLI MOCO and reproducible myocardial T1 measurements, with substantially reduced BH duration while maintaining good image quality. These findings support further evaluation of rapid T1FLASH as an alternative T1 mapping approach in patients with limited BH capacity. Conventional T1 MOLLI MOCO remains the preferred technique when adequate BH can be achieved.

**Supplementary Information:**

The online version contains supplementary material available at https://doi.org/10.1186/s12872-026-06687-8.

## Background

Quantitative mapping of myocardial T1 relaxation times is a well-established diagnostic tool in cardiovascular magnetic resonance imaging (CMR). It enables the non-invasive assessment of regional myocardial tissue composition and is therefore highly relevant in the diagnosis of many cardiovascular diseases e.g., myocarditis, acute myocardial infarction, various cardiomyopathies and congenital heart disease [1–5]. In a wide range of clinical conditions, including focal myocarditis and acute myocardial infarction, regional changes in T1 relaxation measurements are diagnostically relevant in addition to global T1 values [3, 6].

The Modified Look-Locker inversion recovery (MOLLI) sequence is currently the most widely used in everyday clinical practice due to its high precision and reproducibility [3, 7, 8]. MOLLI facilitates pixel-wise T1 estimation through a series of inversion recovery images acquired during breath-holding (BH) [3, 7, 8]. The current motion control algorithms that are implemented in the T1 MOLLI MOCO sequence used in clinical practice are only able to control in-plane motion [9]. Through-plane motion caused by free-breathing (FB) cannot be sufficiently compensated for and still requires long BH maneuvers to obtain good image quality and valid T1 relaxation times [9]. Besides inversion-recovery-based approaches such as T1 MOLLI, saturation-recovery techniques including SASHA have been developed to improve T1 accuracy and reduce heart-rate dependence [4]. However, these techniques generally provide lower precision and signal-to-noise ratio than T1 MOLLI [10]. Consequently, T1 MOLLI remains the most widely used clinical applicable technique for native myocardial T1 mapping.

In current clinical practice, respiratory motion often interferes with myocardial T1 mapping, as many patients are unable to sustain adequate BH e.g., pediatric patients and patients with cardiovascular or pulmonary disease. Respiratory motion may impair myocardial T1 quantification by introducing motion artifacts and image misregistration, leading to inaccurate T1 measurements [11, 12].

Recent advances in real-time MRI enable high-resolution myocardial T1 mapping using a rapid single-shot inversion-recovery fast low-angle shot (T1FLASH) sequence with radial undersampling and regularized iterative reconstruction [13]. The technique is based on a single inversion-recovery acquisition with retrospective selection of diastolic data after global inversion, differing from MOLLI in both inversion-recovery sampling and readout strategy. This approach allows substantially shorter BH durations [5, 13], which may be advantageous in patients with limited BH capacity or during exercise or pharmacological stress.

This study was designed to compare two myocardial T1 mapping approaches in healthy adult volunteers during BH and FB. To our knowledge, no previous study has performed a comprehensive comparison of rapid T1FLASH and the clinically implemented conventional T1 MOLLI MOCO under both BH and FB conditions while systematically investigating the regional effects of respiratory motion on myocardial T1 values. Therefore, the aim of this study was to compare both techniques with respect to quantitative agreement, reproducibility, image quality, participant comfort, and the influence of respiration on global and regional myocardial T1 values. In addition, regional analyses were performed to identify myocardial segments that remain robust to respiratory motion and may provide reliable measurements even when BH is suboptimal.

## Methods

### Study population

The study is a prospective, observational, in vivo cardiac MRI study in healthy, adult volunteers at the University Hospital Düsseldorf. A total of 14 healthy volunteers were included in the study. Participants were recruited on a voluntary basis within the local university community, primarily among medical students. Inclusion criteria were age ≥ 18 years and the absence of known cardiovascular disease. Exclusion criteria included any contraindication to MRI e.g., pregnancy, ferromagnetic implants, severe claustrophobia, and known cardiovascular disease including cardiac implantable electronic devices. Volunteers provided written informed consent prior to participation. The study was approved by the local ethics committee (Ethics Committee of the Medical Faculty, University hospital Düsseldorf, study number 2023–2336) and conducted in accordance with the principles of the Declaration of Helsinki. The study was designed as an exploratory feasibility study; therefore, no formal sample size or power calculation was performed.

### Cardiovascular magnetic resonance imaging

The MRI measurements were performed on a clinical 1.5 T MRI scanner (MAGNETOM Avanto fit, Siemens Healthineers, Erlangen, Germany; software version syngo MR E11) with a 32-channel spine matrix coil combined with an 18-channel body coil in the supine position. Cardiac MRI was performed using a standard cardiac imaging protocol with cardiac localizers and electrocardiogram (ECG)-gated balanced steady-state free precession (bSSFP) sequences to acquire two- and four-chamber cine stacks in end-expiratory BH. These were used for biplanar functional analysis and planning of basal, midventricular and apical slices for T1 mapping. T1 mapping was performed using a commercially available modified Look-Locker inversion recovery (MOLLI) sequence [8] with typical imaging parameters (see Table 1) and a total acquisition of 11 heartbeats in a basal, midventricular and apical slice during end-expiratory BH. The T1 MOLLI MOCO sequence has an integrated algorithm for in-plane motion correction based on the algorithm described by Xue et al. [9]. Thereafter, the T1 mapping was performed using a rapid T1FLASH sequence as previously described [13]. This approach employs a single-shot inversion-recovery FLASH acquisition with radial undersampling and regularized iterative reconstruction [13]. The non-selective adiabatic 180° inversion pulse was triggered to early diastole in order to capture the important early parts of the inversion recovery curve during diastolic phases with almost no motion. After the inversion pulse, a continuous radial FLASH readout was performed without further ECG-triggered interruptions. The ECG signal was recorded throughout the acquisition and used retrospectively to delete systolic frames. The resulting motion-minimized image series was then subjected to pixel-wise fitting of the inversion recovery curve to generate the T1 map [13]. Detailed sequence parameters are provided in Table 1.

**Table 1:** Detailed Sequence Parameters

sequence parameters	T1 MOLLI MOCO	Rapid T1FLASH
sequence family	inversion recovery Look-Locker bSSFP readout (MOLLI)	inversion recovery spoiled gradient echo (IR-FLASH)
motion correction	retrospective non-rigid, in plane	-
TR/TE (ms)	2.82/1.20	2.50/1.72
FOV (mm²)	320 × 320	320 × 320
in-plane resolution (mm²)	1.3 × 1.3	1.4 × 1.4
slice thickness (mm)	9	9
flip angle (°)	35	6
bandwidth (Hz/pixel)	1085	1590
k-space trajectory	Cartesian	Radial, golden angle trajectory (20,89°)
projections	-	15
reconstruction	Fourier + parallel imaging (GRAPPA, acceleration factor 2)	non-linear model-based from radial k-space
duration (s)	11–12	3–4
ECG trigger	prospective	prospective
trigger delay time (ms)	504	400

*ECG* electrocardiogram, *FLASH* fast low angle shot, *FOV* field of view, *MOLLI* modified Look-Locker inversion recovery, *TE* echo time, *TR* trigger time

During the first examination, both T1 MOLLI MOCO and rapid T1FLASH were acquired during end-expiratory BH. Subsequently, both sequences were repeated during FB. Within each breathing condition, the order of the two sequences was randomized across subjects to minimize potential order effects. The breathing conditions themselves were not randomized. Respiratory flow and volume were recorded throughout the FB acquisitions using MR-compatible spirometry as described previously [14, 15].

Following completion of the first examination, participants were completely removed from the MRI scanner, completed the comfort questionnaire, and were given a break of at least 30 min. They were then repositioned and re-examined to assess interstudy reliability. During this second examination, only the BH acquisitions of T1 MOLLI MOCO and rapid T1FLASH were repeated, with the sequence order again randomized across subjects.

### Data analysis

The biplanar LAX function module and the T1 mapping module of the commercially available evaluation software cvi42 (Release 5.17.1.(3504); Circle Cardiovascular Imaging Inc. Calgary, Canada) were used for volumetric analysis and evaluation of the T1 mapping. The LV epicardial and LV endocardial contours and the superior and inferior short-axis reference points were generated automatically by the software. As myocardial blood pool contours are not automatically generated on T1 maps, they were manually delineated on the most basal slice to minimize partial volume effects and avoid interference from papillary muscles. The automatic correction of the epicardial and endocardial contours for movement was set to 20% in the T1 mapping. All T1 analyses were performed by two raters (L.R.: medical doctor and radiology research assistant with 5 years of experience in cardiovascular MRI and Y.H.: medical doctoral candidate) to assess interrater reliability. Rater 1 (L.R.) evaluated the T1 MOLLI MOCO and rapid T1FLASH mapping during BH twice with an interval of at least 4 weeks to test for intrarater reliability.

### Image quality assessment

The image quality of T1 MOLLI MOCO and rapid T1FLASH maps during BH and FB was rated by two raters (L.R. and Y.H.) based on 5-point scales modified from Kellman et al. [16]. The visibility of the LV endocardial contour, the LV epicardial contour, the sharpness of the right ventricular wall and the diagnostic confidence was scored on an ordinal scale (1 = non-diagnostic, 2 = poor, 3 = fair, 4 = good and 5 = excellent), (see Additional file 1: Supporting Information Figure S1). Diagnostic confidence was defined as the readers’ subjective confidence that the T1 map was of sufficient quality for reliable clinical interpretation and myocardial tissue characterization. In addition, the presence of motion artefacts that led to the formation of a myocardial cap and the loss of myocardial integrity was rated from 1 = strongly pronounced, 2 = moderately pronounced, 3 = slightly pronounced, 4 = hardly pronounced, 5 = not present at all (Additional file 1: Supporting Information Figure S1). Because of the characteristic appearance of the reconstructed T1 maps, including background suppression in rapid T1FLASH and visible background noise in T1 MOLLI MOCO, blinding of the readers to the acquisition method was not feasible. However, a blinded assessment was performed regarding the breathing condition (BH vs. FB). Rater 1 (L.R.) reevaluated the image quality with an interval of at least 4 weeks to assess intrarater reliability.

### Quantitative edge sharpness assessment

Myocardial edge sharpness was additionally assessed in the midventricular slices of T1 MOLLI MOCO and T1FLASH during BH quantitatively using the approach described by Ahmad et al. [17] and applied before by Wang et al. [18]. A line profile was drawn from the LV blood pool to the middle of the interventricular septum on the midventricular T1 map. The resulting T1 profile was fitted to a four-parameter sigmoid function, S(x) = a/[1 + exp(− k(b − x))] + c, using nonlinear least-squares fitting with the Levenberg-Marquardt algorithm [Ahmad et al.]. Edge sharpness was quantified as ∣k∣ in mm − 1, with higher values indicating sharper myocardial borders.

### Comfort survey

After the cardiovascular MRI examination, all subjects were surveyed regarding their overall comfort, noise and BH time during T1 MOLLI MOCO and rapid T1FLASH using a standardized questionnaire modified from Chen et al. 2019 [19]. The items were rated on a scale of 1–6 with 1 = “very comfortable” and 6 = “intolerable”.

### Statistical analysis

All statistical analyses were performed using SPSS (IBM SPSS Statistics for Windows, version 29.0.2.0; IBM Corp., Armonk, NY, USA).

The predefined error margin was set at ± 4% of the global mean T1 value during BH to assess diagnostically significant differences in global T1 relaxation times between healthy subjects and the variation in regional T1 relaxation times (values from 16 AHA segments) between BH and FB. This threshold was chosen as a pragmatic agreement criterion within the reported measurement variability of native myocardial T1 mapping (approximately 2.5-5%) [20–25], including published interstudy coefficients of variation of around 4% [26]. Thereby reflecting the expected measurement uncertainty of established T1 mapping methods while remaining sufficiently stringent to detect clinically meaningful differences in myocardial tissue characterization [20–25].

Data distribution was assessed using the Shapiro-Wilk test due to the small sample size. For normally distributed data, paired-samples t-tests were used to compare T1 relaxation times obtained with T1 MOLLI MOCO and rapid T1FLASH during end-expiratory BH and FB, and edge sharpness between the two techniques during BH. For each AHA segment, a one-sided paired t-test was performed to test whether the mean absolute difference between FB and BH measurements was below the predefined and above justified clinically relevant threshold of 4% of the global BH T1 relaxation time across the 14 subjects.

For ordinally scaled data, such as image quality ratings and comfort questionnaire results, the Wilcoxon signed-rank test was applied.

Agreement between measurements obtained with T1 MOLLI MOCO and rapid T1FLASH, as well as between BH and FB acquisitions, was evaluated using Bland-Altman analysis (mean difference and 95% limits of agreement) [27]. Linear correlations between T1 relaxation times were assessed using Pearson correlation coefficients.

Intrarater, interrater, and interstudy reliability for T1 relaxation time measurements were assessed using the intraclass correlation coefficient (ICC) based on a two-way random-effects model with absolute agreement. ICC values were interpreted as excellent (> 0.90), good (> 0.75- ≥0.90), moderate (> 0.50- ≥0.75), or poor (≤ 0.50) [28].

For ordinal data, such as image quality assessments, intra- and interrater reliability were additionally evaluated using Spearman’s rank correlation coefficient (ρ). According to Cohen, correlation coefficients were interpreted as small (0.1–0.3), moderate (0.3–0.5), or strong (> 0.5) [29].

All tests were two-sided, except for the one-sided paired t-test for the analysis of the respiratory-dependent changes across the AHA segments, and a *p*-value < 0.05 was considered statistically significant for the results of the image quality analysis, physiological data and image quality assessment. For the multiple comparisons of the T1 relaxation times (global, basal, apical and midventricular) adjustment for multiple testing was applied to control the family-wise error rate using the Bonferroni method. The nominal significance level (α = 0.05) was divided by the number of comparisons (*n* = 4), resulting in an adjusted significance threshold of α = 0.0125. Accordingly, results were considered statistically significant if the corresponding *p*-value was below this adjusted threshold.

## Results

### Study population

Fourteen healthy young adult volunteers (male = 6, female = 8) were included in the study. None of the volunteers had a history of cardiovascular disease. The volunteers’ ages ranged from 22 to 34, with an average age of 27 ± 4 years, an average body weight of 68 ± 9 kg, and an average body height of 171 ± 8 cm. Body surface area (BSA) was calculated at 1.8 ± 0.1 using the DuBois formula and was used to calculate indexed volumes. The mean heart rate during initial T1 relaxation time acquisition was 65 ± 8 bpm. During the reproducibility study the mean heart rate was slightly, but significantly lower (62 ± 9 bpm; *p* < 0.002). For details on individual participant characteristics, see Table 2.

**Table 2:** Participant Characteristics

no.	sex	age	body weight (kg)	body height (cm)	heart frequence (bpm) (initial study)	heart frequence (bpm) (reproducibility study)	tidal volume indexed (ml/kg)	respiratory rate (1/min)	LV-EDVi (ml/m²)	LV-ESVi (ml/m²)	LV-SVi (ml/m²)	LV-EF (%)
1	male	24	80	178	72	69	4	13	99	37	62	63
2	female	24	61	170	59	57	9	19	84	30	54	65
3	female	24	61	168	66	65	8	16	86	35	51	59
4	female	31	77	162	81	79	5	21	78	23	55	71
5	female	30	60	164	69	59	13	8	94	33	60	64
6	male	31	66	157	61	58	11	12	119	37	82	69
7	male	27	80	176	51	49	19	7	112	44	68	61
8	female	23	57	173	76	73	8	17	80	30	50	62
9	female	25	62	170	67	66	13	16	73	21	51	71
10	male	34	74	181	56	53	14	7	116	44	72	62
11	female	25	68	174	69	64	12	9	81	29	52	65
12	male	29	81	189	59	45	4	16	109	46	63	58
13	female	23	55	167	75	65	12	11	81	23	59	72
14	male	22	72	170	59	59	8	15	79	28	52	65

*bpm*  beats per minute, *LV-EDVi*  left ventricular end-diastolic volume indexed to body surface area, *LV-EF*  left ventricular ejection fraction, *LV-ESVi*  left ventricular end-systolic volume indexed to body surface area, *LV-SVi*  left ventricular stroke volume indexed to body surface area

### MR-compatible spirometry

The time of BH during the conventional MOLLI T1 mapping was 12–14 s per slice. In contrast, rapid T1FLASH required a BH duration of 3–4 s per slice for data acquisition. All healthy volunteers breathed evenly during the FB examination. The respiratory rate was 13 ± 4 per minute and the indicated respiratory volume was 10 ± 4 ml/kg, corresponding to a respiratory minute volume of 121 ± 38 ml/kg/min (Table 2).

### Acquisition and postprocessing workflow

The acquisition time corresponded to the reported BH duration and was 3–4 s for rapid T1FLASH and 12–14 s for T1 MOLLI MOCO. Inline reconstruction of the T1 maps was completed within approximately 2–3 s for both rapid T1FLASH and T1 MOLLI MOCO, allowing immediate availability of the parametric maps after image acquisition.

All contours except the myocardial blood pool contour in the T1 mapping were drawn automatically in cvi42. Manual corrections were not needed. The analysis of the rapid T1FLASH and T1 MOLLI MOCO maps and the analysis of the biplanar function lasted approximately 15 min per volunteer.

### Image quality assessment of T1 mapping

**Fig. 1:**
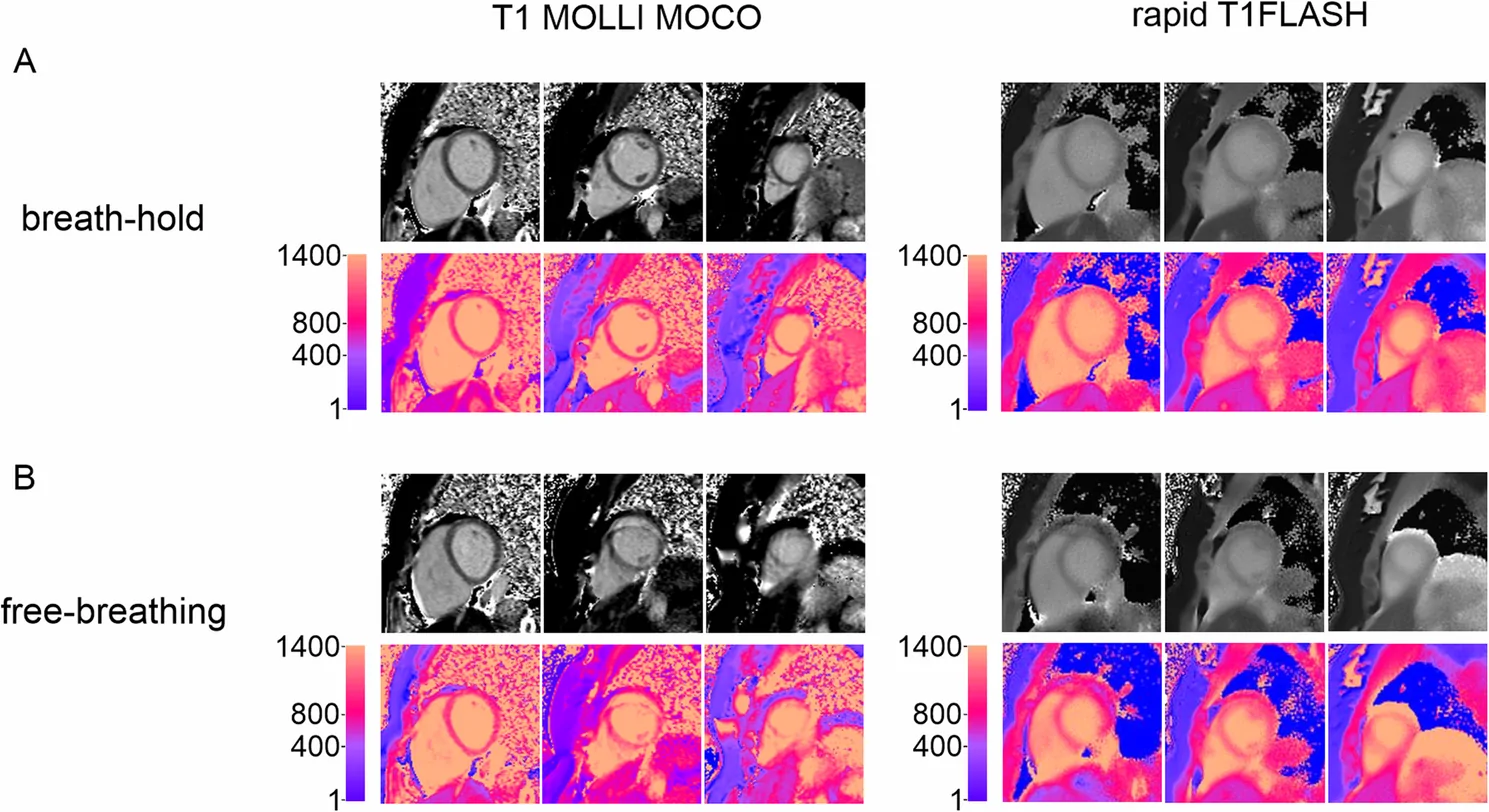
Image Quality during Breath-Hold and Free-Breathing. T1 maps acquired with T1 MOLLI MOCO and rapid T1FLASH during breath-hold showed good to excellent image quality (**A**). During free-breathing, T1 mapping was impaired by motion artifacts for both T1 MOLLI MOCO and rapid T1FLASH (**B**). FLASH = fast low angle shot; MOCO = motion correction; MOLLI = modified Look-Locker inversion recovery

Image quality was rated as good to excellent for both T1 MOLLI MOCO and rapid T1FLASH during BH acquisitions, with T1 MOLLI MOCO receiving marginally, but significantly higher overall scores (T1 MOLLI MOCO: 4.4 ± 0.3; rapid T1FLASH: 4.2 ± 0.3; *p* = 0.02). A detailed summary of ratings for individual quality criteria is provided in Additional file 1 (Additional file 1: Supporting Information Table S1a, b). An exemplary set of T1 maps obtained with T1 MOLLI MOCO and rapid T1FLASH is presented in Fig. 1A.

During FB acquisitions, image quality scores were significantly reduced for both techniques [T1 MOLLI MOCO: BH: 4.4 ± 0.3, FB: 3.1 ± 0.9; (*p* < 0.01); rapid T1FLASH: BH: 4.2 ± 0.3, FB: 2.8 ± 0.8 (*p* < 0.01)]. No statistically significant difference was found between the overall image quality of T1 MOLLI MOCO and rapid T1FLASH during FB (*p* > 0.05) (for details see Additional file 1: Supporting Information Table 1a, b). Representative T1 maps from a subject with marked respiratory motion artifacts, including myocardial capping and loss of myocardial integrity, are shown in Fig. 1B.

### Quantitative edge sharpness assessment

Quantitative analysis confirmed the visually observed difference in myocardial border sharpness. Edge sharpness was significantly lower for rapid T1FLASH than for T1 MOLLI MOCO (1.15 ± 0.19 vs. 1.63 ± 0.29 mm-1, respectively; *p* < 0.01) in the midventricular slices during BH.

### Biplanar function

The biplanar function confirmed normal left ventricular (LV) function parameters and normal dimensions for female and male volunteers (mean LV enddiastolic volume indexed to BSA: male: 106 ± 12 ml/m²; female: 82 ± 6 ml/m², mean LV endsystolic volume indexed to BSA: male: 39 ± 6 ml/m²; female: 28 ± 5 ml/m², mean LV stroke volume indexed to BSA: male: 66 ± 9 ml/m²; female: 54 ± 4 ml/m², LV ejection fraction: male: 63 ± 3%, female: 66 ± 4%, LV diastolic myocardial mass indexed to BSA: male: 64 ± 7 g/m², female: 46 ± 6 g/m²) (Table 2).

### T1 mapping

#### Global analysis during BH

The global T1 relaxation times measured with rapid T1FLASH were significantly longer than those obtained with T1 MOLLI MOCO during BH (Table 3). This difference was also observed for the mean T1 values in the apical slice. The T1 relaxation times in the midventricular and basal slices were not significantly different (*p* > 0.0125) (Table 3). The coefficient of variation was 3.3% for rapid T1FLASH and 2.6% for T1 MOLLI MOCO (Table 3).

**Table 3:** Global T1 MOLLI MOCO and Rapid T1FLASH Relaxation Times

region	breathing	method	mean ± SD (ms)	paired sampled t-test	Pearson correlation
global	breath-holding	T1 MOLLI MOCO	1000 ± 26	*p* < 0.01	*r* = 0.63, *p* < 0.01
		rapid T1FLASH	1030 ± 35		
basal	breath-holding	T1 MOLLI MOCO	1004 ± 29	*p* = 0.02	*r* = 0.67, *p* < 0.01
		rapid T1FLASH	1023 ± 40		
mid-ventricular	breath-holding	T1 MOLLI MOCO	1003 ± 27	*p* = 0.03	*r* = 0.51, *p* = 0.03
		rapid T1FLASH	1022 ± 36		
apical	breath-holding	T1 MOLLI MOCO	989 ± 38	*p* < 0.01	*r* = 0.12, *p* = 0.34
		rapid T1FLASH	1049 ± 44		
global	breath-holding	T1 MOLLI MOCO	1000 ± 26	*p* = 0.03	*r* = 0.51, *p* = 0.03
	free-breathing		1026 ± 50		
basal	breath-holding	T1 MOLLI MOCO	1004 ± 29	*p* = 0.06	*r* = 0.88, *p* < 0.01
	free-breathing		1016 ± 46		
mid-ventricular	breath-holding	T1 MOLLI MOCO	1003 ± 27	*p* = 0.07	*r* = 0.46, *p* = 0.05
	free-breathing		1027 ± 59		
apical	breath-holding	T1 MOLLI MOCO	989 ± 38	*p* = 0.03	*r* = 0.33, *p* = 0.13
	free-breathing		1043 ± 104		
global	breath-holding	rapid T1FLASH	1030 ± 35	*p* < 0.01	*r* = 0.79, *p* < 0.01
	free-breathing		1069 ± 68		
basal	breath-holding	rapid T1FLASH	1023 ± 40	*p* < 0.01	*r* = 0.72, *p* < 0.01
	free-breathing		1066 ± 69		
mid-ventricular	breath-holding	rapid T1FLASH	1022 ± 36	*p* = 0.01	*r* = 0.77, *p* < 0.01
	free-breathing		1064 ± 81		
apical	breath-holding	rapid T1FLASH	1049 ± 44	*p* = 0.03	*r* = 0.33, *p* = 0.02
	free-breathing		1082 ± 66		

*FLASH*  fast low angle shot, *MOCO*  motion correction, *MOLLI*  modified Look-Locker inversion recovery, *SD*  standard deviation

The two techniques showed a strong and significant correlation for both global and basal slice T1 relaxation times (Table 3). A moderate but significant Pearson correlation coefficient was observed in the midventricular slice (Table 3), whereas no significant correlation was found in the apical slices. The apical slices exhibited the highest standard deviations for T1 relaxation times with both rapid T1FLASH and T1 MOLLI MOCO (Table 3).

**Fig. 2:**
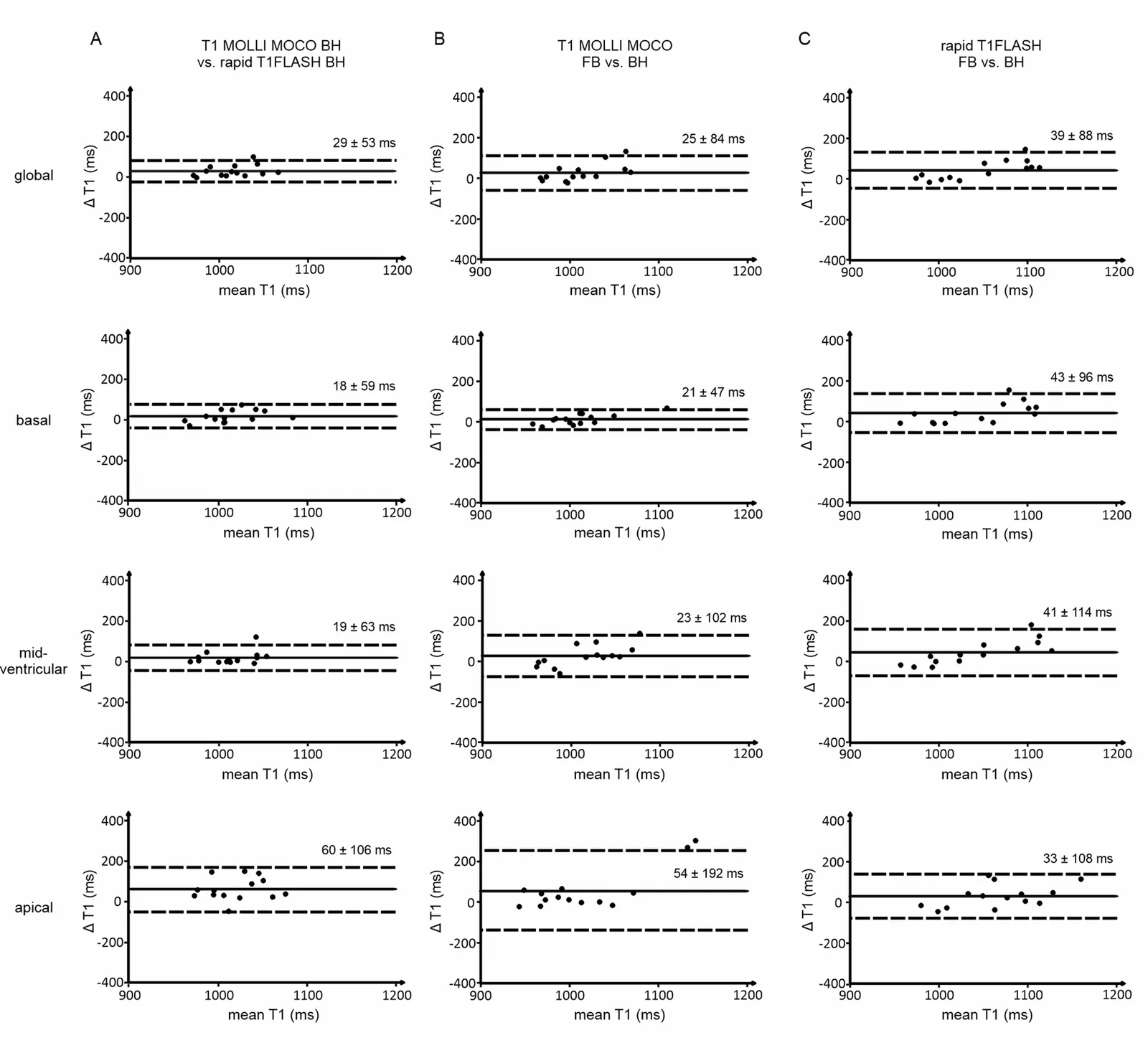
Bland-Altman Analysis. Bland-Altman plots showing global T1 relaxation times and basal, midventricular, and apical slice values comparing T1 MOLLI MOCO and rapid T1FLASH during breath-hold (**A**), T1 MOLLI MOCO during breath-hold and free-breathing (**B**), and rapid T1FLASH during breath-hold and free-breathing (**C**). Δ = difference (MOLLI MOCO vs. FLASH / breath-hold – free-breathing); solid line = mean value; dashed lines = ± 1.96 standard deviations. FLASH = fast low-angle shot; MOCO = motion correction; MOLLI = modified Look-Locker inversion recovery

Agreement between global T1 MOLLI MOCO and rapid T1FLASH values during BH was assessed using Bland-Altman plots (Fig. 2). For the comparison between T1 MOLLI and rapid T1 FLASH acquired during BH (Fig. 2A), the mean bias for global myocardial T1 values was 29 ± 53 ms (mean bias ± 1.96 SD, corresponding to the 95% limits of agreement). Similar biases were observed in the basal and mid-ventricular slices (18 ± 59 ms and 19 ± 63 ms, respectively), whereas the apical slice showed a larger bias and wider limits of agreement (60 ± 106 ms).

#### Global analysis during FB

During FB, global T1 relaxation times as well as basal, midventricular, and apical slices values were numerically higher than the corresponding times obtained with both rapid T1FLASH and T1 MOLLI MOCO during BH (Table 3). The mean change in global myocardial T1 values between BH and FB acquisitions was numerically larger for rapid T1FLASH than for T1 MOLLI MOCO (Table 3); however, this difference between techniques was not statistically significant (*p* = 0.2). A paired-samples t-test confirmed that the difference between FB and BH was statistically significant within rapid T1FLASH for the global T1 relaxation times in the basal and midventricular slice (*p* < 0.0125) (Table 3).

Overall, both methods demonstrated diagnostically relevant higher standard deviations during FB for the mean global T1 relaxation time and the basal, midventricular, and apical slices. Pearson correlation coefficient revealed a significant moderate-to-strong correlation between T1 relaxation times obtained with T1 MOLLI MOCO and rapid T1FLASH both during BH and FB, except for the apical slices (Table 3).

A detailed description of the agreement between global T1 MOLLI MOCO and rapid T1FLASH values during both BH and FB is provided using Bland-Altman plots (Fig. 2). Comparison of T1 MOLLI MOCO acquired during FB and BH (Fig. 2B) demonstrated a mean bias of 25 ± 84 ms for global myocardial T1 values. Regional analyses revealed biases of 21 ± 47 ms in the basal slice and 23 ± 102 ms in the mid-ventricular slice. The apical slice exhibited the greatest variability, with a mean bias of 54 ± 192 ms (mean bias ± 1.96 SD, corresponding to the 95% limits of agreement).

For rapid T1FLASH, the variability between FB and BH acquisitions was likewise high (Fig. 2C). The mean bias for global myocardial T1 values was 39 ± 88 ms, with biases of 43 ± 96 ms, 41 ± 114 ms, and 33 ± 108 ms in the basal, mid-ventricular, and apical slices (mean bias ± 1.96 SD, corresponding to the 95% limits of agreement).

#### Regional analysis during BH and FB

**Fig. 3:**
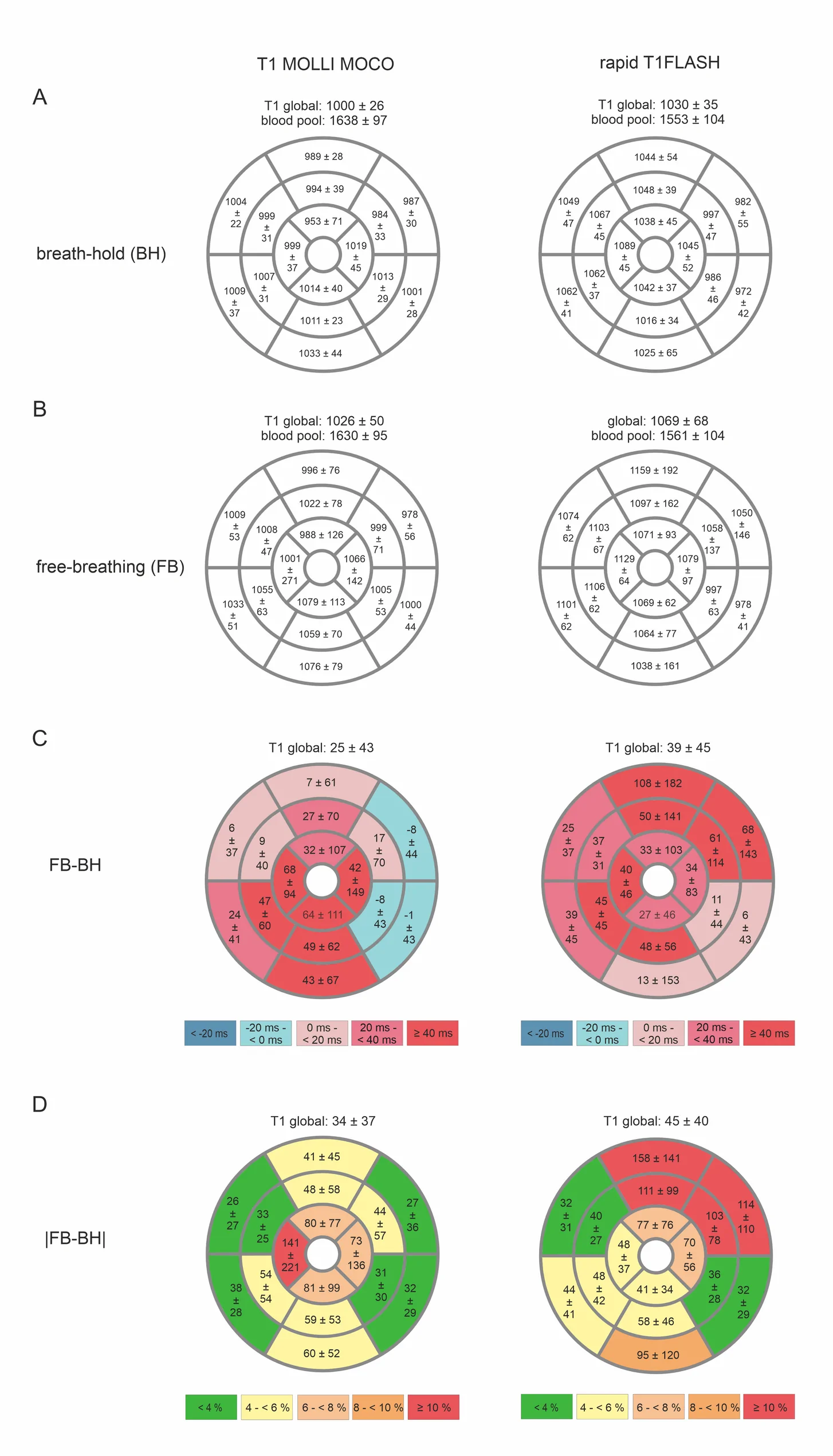
Regional T1 Relaxation Times during Breath-Hold and Free-Breathing. Regional T1 relaxation times of AHA segments and the global T1 relaxation time for T1 MOLLI MOCO and rapid T1FLASH during breath-hold (BH) (**A**) and free-breathing (FB) (**B**). Differences between T1 MOLLI MOCO and rapid T1FLASH during BH and FB are shown in part (**C**). Most AHA segments exhibited a predominant increase in T1 relaxation time during FB except of AHA segments 5 (basal inferolateral), 6 (basal anterolateral), and 12 (mid anterolateral) in T1 MOLLI MOCO (**C**) Absolute mean differences between FB and BH were less affected by respiratory artefacts for both techniques in AHA segments 2 (basal anteroseptal), 5 (basal inferolateral), 8 (mid anteroseptal), and 11 (mid inferolateral) (**D**). AHA = American Heart Association, BH = breath-hold, FLASH = fast low angle shot, FB = free-breathing, MOLLI = modified Look-Locker inversion recovery

The mean regional T1 values across the 16 AHA segments for T1 MOLLI MOCO and rapid T1FLASH during BH are presented in Fig. 3a (Fig. 3A). During FB, relaxation times were longer in most of the AHA segments and most of the participants (Fig. 3B). However, in some volunteers selected segments showed shorter T1 relaxation times, leading to overall reduced values in segments 5 and 6 with T1 MOLLI MOCO during FB (see Fig. 3B). Overall, variability of segmental T1 measurements increased significantly with apical segments being more and AHA segments 2 (basal anteroseptal), 5 (basal inferolateral), 8 (mid anteroseptal), 11 (mid inferolateral) less affected by respiratory artefacts in both techniques (see Fig. 3C, D for details).

### Reproducibility

The ICC demonstrated good to excellent reproducibility for interrater reliability of the T1 relaxation times acquired with T1 MOLLI MOCO and rapid T1FLASH during BH and FB and for the intrarater and interstudy reliability during BH (see Table 4 for details). Only the interrater reliability of apical T1 measurements obtained with T1 MOLLI MOCO during FB was classified as moderate based on the ICC.

**Table 4:** Reproducibility

T1 relaxation times		T1 MOLLI MOCO				Rapid T1FLASH			
		global	basal	mid-ventricular	apical	global	basal	mid-ventricular	apical
Interrater reliability (ICC)	BH	1.00	1.00	1.00	1.00	1.00	1.00	0.99	1.00
	FB	1.00	1.00	1.00	1.00	0.99	1.00	1.00	0.99
Intrarater reliability (ICC)	BH	0.99	1.00	1.00	0.90	1.00	0.99	1.00	1.00
Interstudy reliability (ICC)	BH	0.95	0.98	0.92	0.61	0.95	0.91	0.93	0.90
Image quality score									
Interrater reliability (Spearman’s rank correlation)	BH	0.93	0.97	0.94	0.97	0.87	0.88	0.80	0.97
	FB	0.92	0.85	0.92	0.68	0.89	0.90	0.98	0.94
Intrarater reliability (Spearman’s rank correlation)	BH	0.97	0.92	0.91	0.99	0.97	0.89	0.89	0.98
	FB	0.88	0.69	0.94	0.92	0.89	0.98	0.90	0.87

*BH*  breath-holding, *FB*  free-breathing, *FLASH*  fast low angle shot, *ICC*  Intraclass correlation coefficient, *MOCO*  motion control, *MOLLI*  modified Look-Locker inversion recovery

Spearman’s rank correlation coefficient showed a strong and statistically significant correlation for the different image quality criteria for inter- and intrarater reliability during FB and BH (Table 3).

### Comfort survey

**Fig. 4:**
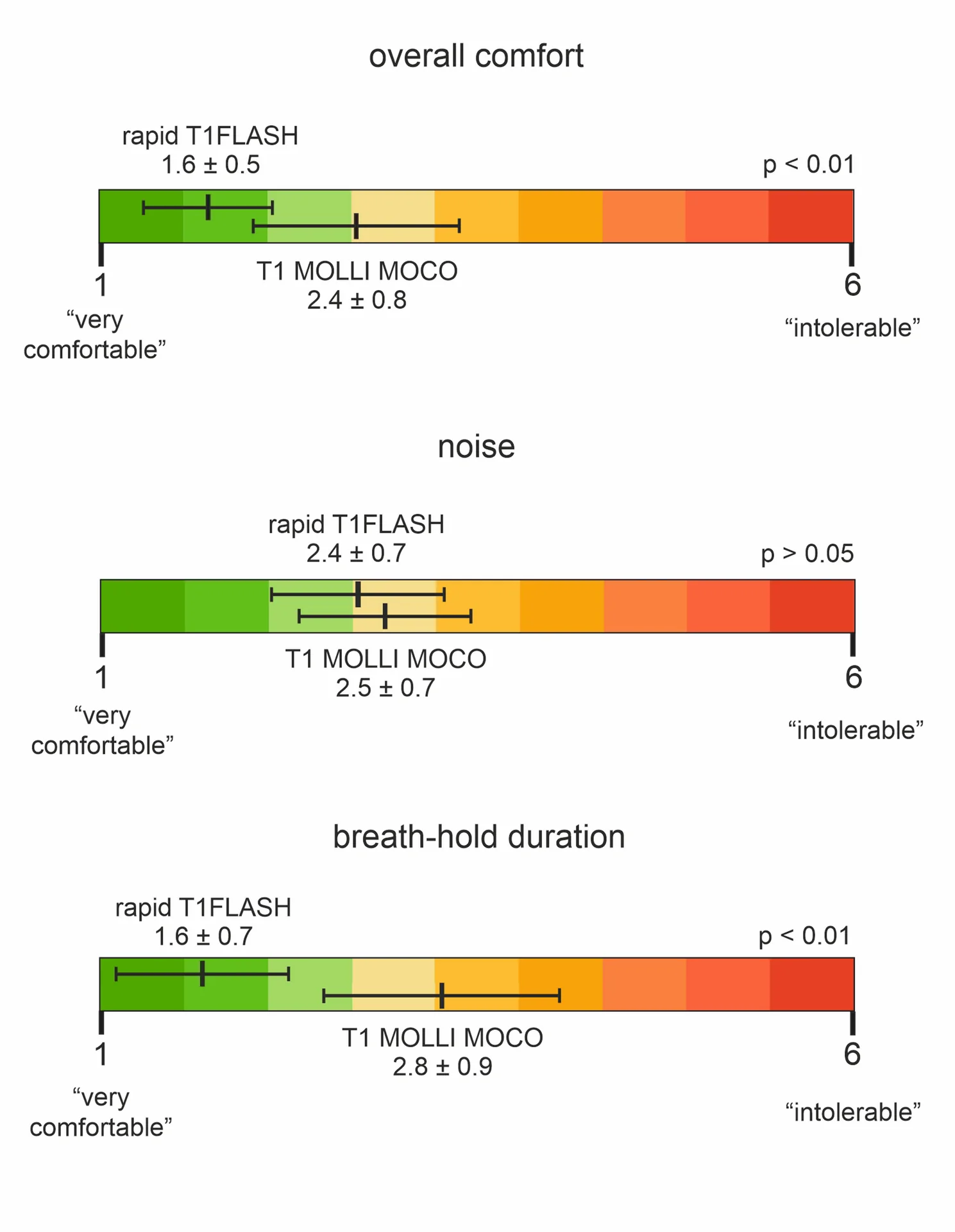
Comfort Scale Results. Mean comfort rating for the items *overall comfort*, *noise* and *breath-hold duration* for T1 MOLLI MOCO and rapid T1FLASH. FLASH = fast low angle shot, MOLLI = modified Look-Locker inversion recovery

Overall comfort was rated significantly lower for T1 MOLLI MOCO compared with rapid T1FLASH (Fig. 4), primarily due to the longer BH duration (Fig. 4).

## Discussion

Our study addressed two major aspects, which warrant separate discussions. First, we compared myocardial T1 mapping using the widely used motion-corrected MOLLI sequence to a novel rapid T1FLASH sequence [5, 13] during BH. Second, we systematically analyzed the influence of respiration on global and regional myocardial T1 relaxation times in both sequences. To our knowledge, this is the first study to comprehensively compare rapid T1FLASH and conventional T1 MOLLI MOCO under both BH and FB conditions. Beyond quantitative agreement, the study also evaluated reproducibility, image quality, participant comfort, and the regional effects of respiratory motion, providing a comprehensive assessment of the practical performance of both techniques.

The comparison between conventional T1 MOLLI MOCO and the novel rapid T1FLASH sequence demonstrated good agreement for global native T1 values obtained with both techniques in healthy volunteers. The coefficient of variation was only slightly higher with rapid T1FLASH compared with T1 MOLLI MOCO (SD of global native T1: 3.3% vs. 2.6%), proving the technical feasibility of rapid T1FLASH. This slightly increased variability is also consistent with the intrinsic signal characteristics of the underlying readout. The spoiled gradient-echo acquisition used in rapid T1FLASH is inherently less SNR-efficient than the bSSFP readout employed in conventional MOLLI. In spoiled gradient-echo imaging, residual transverse magnetization is deliberately spoiled after each excitation pulse, whereas bSSFP maintains transverse coherence across successive repetition intervals, thereby providing substantially higher signal efficiency under otherwise comparable acquisition conditions. Consequently, FLASH-based acquisitions are expected to exhibit slightly increased voxel-wise signal fluctuations, resulting in a greater standard deviation and coefficient of variation within a myocardial region of interest. Previous studies comparing FLASH- and bSSFP-based myocardial T1 mapping have likewise acknowledged the lower intrinsic signal efficiency of FLASH while demonstrating that accurate and reproducible T1 estimation can nevertheless be achieved using appropriate signal modeling and fitting approaches [30]. Despite this expected reduction in SNR efficiency, the increase in variability observed in our study was modest (3.3% vs. 2.6%), and reproducibility remained good to excellent. This suggests that the lower intrinsic SNR of the FLASH readout did not substantially impair the robustness of myocardial T1 quantification in healthy volunteers. Instead, the shorter acquisition time and reduced sensitivity of spoiled gradient-echo imaging to motion-related phase inconsistencies may partly compensate for its lower signal efficiency [30, 31]. Rapid T1FLASH thereby demonstrated promising technical performance under the standardized conditions of this study. Further studies involving patients and the definition of reference values in larger cohorts of healthy subjects are required before its clinical utility can be established.

An important consideration when interpreting the results is that the present study compared two complete T1 mapping implementations rather than isolated method components. The rapid T1FLASH approach differs from conventional T1 MOLLI MOCO not only in the underlying spoiled gradient-echo readout, but also in its radial acquisition strategy, shortened acquisition window, iterative nonlinear inverse reconstruction and a single-shot inversion. Likewise, the conventional MOLLI implementation incorporates a Cartesian bSSFP readout and vendor-specific motion correction of data from multiple inversion pulses. Consequently, the observed differences cannot be attributed to a single technical feature. Instead, our results reflect the overall performance of two acquisition and reconstruction pipelines. Future methodological studies evaluating individual acquisition and reconstruction components separately would be valuable to determine their respective contributions to image quality, precision, and robustness.

Rapid T1FLASH yielded slightly higher global and regional T1 relaxation times than T1 MOLLI MOCO. Minor differences in T1 relaxation times using T1 MOLLI MOCO and rapid T1FLASH techniques were already described in previous studies [5, 13]. Although the lower SNR efficiency of the FLASH readout may contribute to slightly increased measurement variability, it is unlikely to explain the systematic difference in measured T1 values. Rather, this small bias can be explained by the presence of residual in-plane and through-plane motion affecting motion-sensitive bSSFP images as acquired at different inversion times using a modified Look-Locker scheme more than the rapid T1FLASH technique [13, 32]. This phenomenon arises from the dependence of bSSFP readouts on maintaining phase coherence over multiple repetition intervals, which is inherently susceptible to be affected by motion. To reduce these artefacts, T1 MOLLI is used by default and also in our study with an implemented motion correction that is typically performed retrospectively at the image level using non-rigid registration to mitigate small in-plane displacements. Nevertheless, it cannot account for through-plane motion and thus still necessitates BH to ensure consistent voxel-wise signal evolution. In contrast, the rapid T1FLASH approach, designed as a rapid spoiled gradient-echo acquisition, does not rely on inter-repetition phase coherence and is substantially less affected by myocardial motion or flow-related disturbances [13, 32]. The in-plane motion correction used in T1 MOLLI does not appear directly transferable to rapid T1 FLASH, as the single-shot acquisition strategy already minimizes inter-image misregistration. In addition, motion correction algorithms may introduce errors in T1 estimation through imperfect image registration and partial mixing of signals from adjacent tissue structures, especially at tissue interfaces, potentially resulting in biased T1 estimates.

The present study focused on comparison of rapid T1FLASH with T1 MOLLI MOCO because it remains the most widely established clinical reference for myocardial T1 mapping. Other approaches, including saturation-recovery techniques such as SASHA, provide improved T1 accuracy and reduced heart-rate dependence but generally at the expense of lower precision and signal-to-noise ratio [4, 10]. Rather than addressing the trade-off between accuracy and precision, rapid T1FLASH primarily aims to improve acquisition efficiency by substantially reducing BH duration while maintaining robust quantitative performance.

Both sequences showed poorer performance apically, reflected by greater variability of T1 values in apical slices. This finding is consistent with previous studies showing increased dispersion of apical T1 values [21, 33, 34]. A well-recognized explanation is the heightened susceptibility of the apical myocardium to partial-volume effects, most notably the inclusion of blood-pool signals into the voxel which affects T1 quantification in this region [21, 33, 34]. This has clinical relevance, as T1 relaxation times are consequently of limited diagnostic value in the apical region and, when considered in isolation, can lead to over- or underdiagnosis of diseases, e.g., in focal myocarditis.

Image quality of rapid T1FLASH during BH was comparable to that of T1 MOLLI MOCO, while offering a significantly reduced BH duration (3–4 s for rapid T1FLASH vs. 12–14 s for T1 MOLLI MOCO). Quantitative edge-sharpness analysis confirmed significantly lower edge sharpness for rapid T1FLASH compared with T1 MOLLI MOCO in midventricular slices during BH. Nevertheless, the endocardial border remained sufficiently well-defined for automated contour detection using commercially available analysis software. A 20% inward offset from the endocardial border was applied for myocardial T1 analysis to minimize potential partial-volume effects from the LV blood pool. Thus, the potential impact of reduced edge sharpness on myocardial T1FLASH relaxation times may have been minimized.

Participant feedback indicated greater comfort with the shorter BH, which can be expected to be beneficial for patients with limited BH capacity, such as pediatric patients or those with cardiopulmonary disease and in T1 mapping during exercise [35].

The second part of our study investigated myocardial T1 mapping under both BH and FB conditions using both sequences. Global and regional myocardial T1 values were analyzed according to the standardized AHA 16-segment model [36].

The global T1 relaxation times across the AHA segments under BH conditions demonstrated comparable and reliable results for both T1 MOLLI MOCO and rapid T1FLASH acquisitions. Beyond this overall agreement, the present study systematically characterized the regional effects of respiratory motion on myocardial T1 mapping. Rather than affecting all myocardial segments equally, FB resulted in distinct regional patterns of T1 over- and underestimation. As expected, global T1 values and image quality deteriorated in a diagnostically relevant way during FB, as both sequences are primarily optimized for BH acquisitions. During FB, T1 relaxation times were predominantly overestimated, although some AHA segments also showed reduced T1 values. Reduced T1 values during FB were observed in AHA segments 5 (basal inferolateral), 6 (basal anterolateral), and 10 (mid inferior), which are largely adjacent to the lung, resulting in decreased relaxation times due to overlap with lung tissue during respiratory motion. The observed regional patterns of over- and underestimation are consistent with previous reports [37, 38]. Consequently, T1 mapping using currently available clinical sequences remains substantially limited during FB. However, the present findings demonstrate that respiratory motion does not affect all myocardial regions equally, providing useful information for the interpretation of T1 maps acquired under non-ideal breathing conditions and for the development of future motion-robust T1 mapping approaches.

The average tidal volume measured by MR-compatible spirometry was approximately 10 ml/kg, slightly above the values typically expected during quiet breathing (~ 7 ml/kg) [39]. This could be explained by the experimental setting and the increased level of agitation or by the multiple BH conditions performed prior to the FB measurement.

Among the two methods, T1 MOLLI MOCO demonstrated superior overall performance during FB. The exact mechanisms underlying this observation remain incompletely understood. The principal distinction between the two approaches is the application of in-plane motion correction in the T1 MOLLI sequence. This framework employs non-rigid image registration using synthetic contrast-matched reference images to enable robust alignment of inversion images despite their markedly different signal intensities [9]. While this approach effectively compensates for residual in-plane motion, it does not correct through-plane motion which is likely to be more pronounced during spontaneous breathing [14, 40]. Therefore, although in-plane motion correction may improve image alignment, it is unlikely to fully explain the superior FB performance observed for T1 MOLLI MOCO. Additional factors, including differences in acquisition timing, reconstruction, and sampling strategy, are also likely to contribute. Specifically, the temporal characteristics of the acquisition schemes could partly explain the observed differences. The end-expiratory phase represents the longest and most stable period within the respiratory cycle. Given the longer acquisition window of T1 MOLLI MOCO, it is plausible that a larger proportion of data is sampled during this relatively quiescent phase, thereby facilitating more robust image reconstruction. In contrast, the considerably shorter acquisition time of the rapid T1FLASH sequence (3–4 s) may result in a higher proportion of data being acquired during phases of rapid respiratory motion, particularly during inspiration and expiration, which could adversely affect image quality.

Although FB led to statistic significant deterioration across all AHA segments, some segments were less affected by respiratory motion (particularly septal and lateral regions). This observation can be attributed to the predominant cardiac motion during respiration along the superior-inferior axis [14, 40]. Consequently, it is plausible that myocardial regions located adjacent to the lung, liver, or diaphragm, i.e., tissues exhibiting markedly different T1 relaxation times, are affected more strongly by respiratory motion, leading to more pronounced respiratory artifacts in these areas whereas septal and lateral regions are less affected.

Even in myocardial regions that appeared less affected by susceptibility artifacts, reliable T1 measurements could not be obtained. Given this observation, careful monitoring of respiration during clinical examinations, for example using an abdominal belt, appears particularly important to avoid misdiagnosis due to respiratory motion-degraded acquisitions.

Future research should focus on developing motion-robust T1 mapping techniques capable of providing accurate quantification during FB. Several research groups are pursuing such approaches [12, 41–44]. In addition, recent developments in motion-resolved cardiovascular MR quantitative imaging techniques retain the heart-rate insensitivity of FLASH mapping and add FB and multi-slice capabilities [45, 46]. These methods have the potential to improve multiparametric imaging in patients with arrhythmia or difficulty holding their breath. Nevertheless, technical limitations e.g., long computation times and the reduced number of trials involving patients have so far precluded their routine clinical use [44–46].

Although rapid T1FLASH was studied only in healthy subjects in this study, the shorter BH time suggests that it might be beneficial to investigate this approach in patients with limited BH capacity (e.g., children or patients with lung diseases). To test this hypothesis, comprehensive studies in patients are needed to validate the diagnostic performance and clinical applicability of rapid T1 FLASH.

In addition to significantly reducing scan time, rapid T1FLASH may offer further clinical benefits that warrant more detailed investigation. In contrast to bSSFP, spoiled gradient-echo imaging is less susceptible to off-resonance effects, which have been shown to introduce T1 estimation errors and regional image artifacts in bSSFP-based myocardial T1 mapping, particularly at higher field strengths [47, 48]. Consequently, rapid T1FLASH may prove valuable in clinical scenarios where bSSFP imaging is challenging, such as in patients with cardiac implantable electronic devices or at 3 T, where B0 inhomogeneity, banding artifacts, and B1-related effects become more pronounced [49]. Previous work has demonstrated the feasibility of FLASH-based myocardial T1 mapping in patients with implantable cardiac devices, supporting this potential clinical application [50]. Although these applications were not investigated in the present study, they may represent promising areas for future clinical evaluations.

### Limitations

This study demonstrates the feasibility of myocardial T1 mapping using a novel rapid T1FLASH sequence with a substantially reduced BH duration compared with T1 MOLLI MOCO. Nevertheless, several limitations should be considered.

The study was conducted in a relatively small cohort of healthy volunteers, limiting the statistical power and generalizability of the findings. Consequently, the performance of rapid T1FLASH in patients with cardiac disease remains to be established. Future studies including patient populations are required to determine whether the sequence provides reliable T1 quantification under pathological conditions and whether its shorter acquisition time translates into a clinical benefit for patients with limited BH capacity. In addition, reference values derived from larger cohorts of healthy subjects will be necessary before rapid T1FLASH can be implemented in routine clinical practice.

The present study compared two T1 mapping approaches rather than isolated technical components. Specifically, the conventional T1 MOLLI MOCO sequence incorporates vendor-implemented motion correction, whereas the investigated rapid T1FLASH implementation does not. This difference should be considered when interpreting the image quality and FB results, as the observed performance reflects the combined effects of the acquisition strategy and the respective reconstruction approaches rather than a single technical feature. Furthermore, the rapid T1FLASH sequence evaluated in this study is not yet widely available on clinical MRI systems, which currently limits its broader clinical applicability.

A further limitation of this study is that subjective image quality assessment could not be performed in a blinded fashion because the two techniques produce visually distinct T1 maps. Rapid T1FLASH applies a reconstruction and masking strategy that suppresses background and lung signal, whereas T1 MOLLI MOCO retains visible background noise in areas with unreliable T1 estimation. Consequently, reader ratings may have been influenced by these inherent differences in image appearance rather than solely by myocardial image quality. Finally, the heart rate was significantly lower during the reproducibility examination, that was performed approximately 30 min after the initial study, which may have influenced the assessment of interstudy reproducibility. However, heart rate remained stable during the consecutive T1 MOLLI MOCO and rapid T1FLASH acquisitions within each scanning session and therefore is unlikely to have affected the direct comparison between the two techniques.

## Conclusions

Rapid T1FLASH as an integrated acquisition and reconstruction framework for a single-shot inversion demonstrated good agreement with conventional T1 MOLLI MOCO and reproducible myocardial T1 measurements, with substantially reduced BH duration while maintaining good image quality. These findings provide the basis for further evaluation of rapid T1FLASH in patients with limited BH capacity. Conventional T1 MOLLI MOCO remains the preferred technique when adequate BH can be achieved.

## Supplementary Information

Additional file 1: Supporting Information Figure S1: Representative T1 maps illustrating different image quality scores for motion-corrected T1 MOLLI (MOCO) and rapid T1FLASH at three short-axis levels (basal, midventricular, and apical). Supporting Information Table S1: Detailed Ratings of Individual Image Quality Criteria for T1 MOLLI MOCO (a) and rapid T1FLASH (b) acquired under Breath-Holding and Free-Breathing.

## Data Availability

The original contributions presented in the study are included in the article/Supplementary Material, further inquiries can be directed to the corresponding author.

## Abbreviations

BH: Breath-hold/ breath-holding
bSSFP: Balanced steady-state free precession
CMR: Cardiovascular magnetic resonance imaging
ECG: Electrocardiogram
FLASH: Fast low-angle shot
FB: Free-breathing
MOLLI: Modified Look-Locker inversion recovery
MRI: Magnetic resonance imaging
MOCO: Motion control
SD: Standard deviation
