# Supplementary material for: Native myocardial T1 mapping using rapid 1.5T T1FLASH and T1 MOLLI during breath-hold and free-breathing
Source: BMC Cardiovasc Disord. 2026 Sep 23;26:842. doi: 10.1186/s12872-026-06687-8 (PMC13613673; doi:10.1186/s12872-026-06687-8)
Supplement: Supplementary file 1 [file 12872_2026_6687_MOESM1_ESM.docx]

Additional file 1

**Supporting Information Figure S1.** Representative T1 maps illustrating different image quality scores for (A) motion-corrected T1 MOLLI (MOCO) and (B) rapid T1FLASH at three short-axis levels (basal, midventricular, and apical). Shown are examples rated with overall image quality scores of 1 (“non-diagnostic”), 3 (“fair”), and 5 (“excellent”) based on visual assessment of myocardial T1 maps and the presence of respiratory artifacts. The color scale represents T1 relaxation times in milliseconds. MOLLI = modified Look-Locker inversion recovery; MOCO = motion correction; FLASH = fast low-angle shot.

**Supporting Information Table S1**. Detailed Ratings of Individual Image Quality Criteria for T1 MOLLI MOCO (a) and rapid T1FLASH (b) acquired under Breath-Holding and Free-Breathing.

| T1 MOLLI MOCO | | visibility of | | | | | | sharpness of RV wall | | | diagnostic  confidence | | | motion artefact | | | | | | mean ± SD |
| --- | --- | --- | --- | --- | --- | --- | --- | --- | --- | --- | --- | --- | --- | --- | --- | --- | --- | --- | --- | --- |
|  |  | endocardial contour | | | epicardial contour | | |  |  |  |  |  |  | myocardial capping | | | loss of integrity | | |  |
| BH | no. | basal | mid-ventricular | apical | basal | mid-ventricular | apical | basal | mid-ventricular | apical | basal | mid-ventricular | apical | basal | mid-ventricular | apical | basal | mid-ventricular | apical |  |
|  | 1 | 5 | 5 | 3 | 5 | 5 | 3 | 3 | 5 | 5 | 5 | 4 | 3 | 5 | 4 | 3 | 5 | 5 | 3 | 4.2 ± 1.3 |
|  | 2 | 5 | 5 | 3 | 4 | 4 | 3 | 4 | 5 | 3 | 4 | 4 | 3 | 3 | 4 | 3 | 4 | 4 | 3 | 3.8 ± 1.1 |
|  | 3 | 5 | 5 | 4 | 5 | 5 | 5 | 3 | 3 | 3 | 5 | 5 | 4 | 5 | 5 | 5 | 5 | 4 | 4 | 4.4 ± 1.2 |
|  | 4 | 4 | 4 | 4 | 5 | 4 | 4 | 3 | 3 | 3 | 4 | 4 | 4 | 5 | 3 | 3 | 5 | 5 | 5 | 4.0 ± 1.2 |
|  | 5 | 5 | 5 | 4 | 5 | 5 | 4 | 4 | 3 | 3 | 5 | 5 | 3 | 5 | 5 | 5 | 5 | 5 | 4 | 4.4 ± 1.2 |
|  | 6 | 4 | 4 | 4 | 5 | 4 | 4 | 5 | 4 | 3 | 5 | 4 | 4 | 5 | 4 | 4 | 4 | 5 | 5 | 4.3 ± 1.1 |
|  | 7 | 5 | 5 | 5 | 4 | 5 | 4 | 4 | 5 | 5 | 4 | 5 | 5 | 4 | 4 | 4 | 4 | 5 | 5 | 4.6 ± 1.1 |
|  | 8 | 5 | 5 | 5 | 4 | 4 | 4 | 4 | 3 | 4 | 4 | 5 | 5 | 5 | 5 | 5 | 4 | 5 | 4 | 4.4 ± 1.2 |
|  | 9 | 5 | 5 | 3 | 5 | 5 | 3 | 4 | 5 | 5 | 5 | 5 | 3 | 5 | 4 | 3 | 5 | 5 | 2 | 4.3 ± 1.4 |
|  | 10 | 5 | 5 | 5 | 5 | 5 | 5 | 3 | 3 | 4 | 5 | 5 | 5 | 5 | 5 | 5 | 5 | 5 | 5 | 4.7 ± 1.2 |
|  | 11 | 5 | 5 | 4 | 5 | 5 | 5 | 3 | 3 | 3 | 5 | 5 | 4 | 5 | 5 | 5 | 5 | 5 | 4 | 4.5 ± 1.2 |
|  | 12 | 5 | 5 | 5 | 5 | 5 | 5 | 5 | 5 | 5 | 5 | 5 | 5 | 5 | 5 | 5 | 5 | 5 | 4 | 4.9 ± 1.1 |
|  | 13 | 5 | 5 | 5 | 5 | 5 | 5 | 4 | 3 | 4 | 5 | 5 | 5 | 5 | 5 | 4 | 5 | 5 | 5 | 4.7 ± 1.2 |
|  | 14 | 5 | 5 | 5 | 5 | 5 | 5 | 4 | 5 | 5 | 5 | 5 | 5 | 5 | 5 | 5 | 5 | 5 | 5 | 4.9 ± 1.1 |
|  | mean ± SD | 4.9  ± 0.3 | 4.9  ± 0.3 | 4.2  ± 0.8 | 4.8  ± 0.4 | 4.7  ± 0.5 | 4.2  ± 0.8 | 3.8  ± 0.7 | 3.9  ± 1.0 | 3.9  ± 0.9 | 4.7  ± 0.5 | 4.7  ± 0.5 | 4.1  ± 0.8 | 4.8  ± 0.6 | 4.5  ± 0.6 | 4.2  ± 0.9 | 4.7  ± 0.5 | 4.9  ± 0.3 | 4.1  ± 0.9 | 4.4 ± 0.3 |
| FB | 1 | 5 | 5 | 3 | 5 | 5 | 4 | 5 | 5 | 5 | 4 | 5 | 3 | 4 | 4 | 3 | 5 | 5 | 4 | 4.1 ± 1.2 |
|  | 2 | 4 | 4 | 3 | 4 | 4 | 3 | 3 | 3 | 3 | 4 | 4 | 3 | 4 | 3 | 3 | 4 | 3 | 3 | 3.3 ± 0.9 |
|  | 3 | 5 | 5 | 4 | 5 | 5 | 3 | 3 | 3 | 3 | 4 | 4 | 4 | 5 | 4 | 4 | 4 | 4 | 4 | 3.9 ± 1.1 |
|  | 4 | 3 | 3 | 4 | 3 | 3 | 3 | 2 | 2 | 2 | 3 | 3 | 4 | 5 | 5 | 5 | 3 | 3 | 4 | 3.3 ± 1.2 |
|  | 5 | 4 | 1 | 1 | 5 | 1 | 2 | 1 | 2 | 2 | 4 | 1 | 1 | 4 | 1 | 1 | 4 | 1 | 1 | 1.9 ± 1.4 |
|  | 6 | 4 | 5 | 5 | 4 | 4 | 4 | 3 | 4 | 4 | 4 | 4 | 4 | 4 | 3 | 3 | 4 | 4 | 4 | 3.7 ± 1.0 |
|  | 7 | 3 | 1 | 3 | 2 | 1 | 1 | 2 | 4 | 4 | 1 | 1 | 3 | 2 | 1 | 2 | 2 | 1 | 3 | 1.8 ± 1.0 |
|  | 8 | 4 | 4 | 5 | 3 | 4 | 2 | 3 | 3 | 3 | 3 | 4 | 4 | 4 | 3 | 3 | 2 | 3 | 4 | 3.3 ± 1.1 |
|  | 9 | 1 | 2 | 2 | 1 | 2 | 3 | 3 | 4 | 4 | 1 | 3 | 1 | 1 | 2 | 2 | 1 | 4 | 2 | 1.9 ± 1.0 |
|  | 10 | 3 | 1 | 1 | 3 | 1 | 2 | 2 | 3 | 3 | 3 | 1 | 1 | 2 | 1 | 1 | 2 | 1 | 1 | 1.6 ± 0.9 |
|  | 11 | 5 | 4 | 3 | 4 | 4 | 3 | 2 | 5 | 5 | 4 | 4 | 3 | 4 | 3 | 3 | 5 | 4 | 3 | 3.5 ± 1.1 |
|  | 12 | 5 | 5 | 4 | 5 | 5 | 5 | 5 | 5 | 5 | 5 | 5 | 4 | 5 | 4 | 4 | 5 | 5 | 4 | 4.4 ± 1.1 |
|  | 13 | 5 | 1 | 5 | 5 | 1 | 4 | 1 | 4 | 4 | 4 | 1 | 4 | 5 | 2 | 2 | 4 | 1 | 4 | 3.1 ± 1.7 |
|  | 14 | 4 | 5 | 4 | 4 | 4 | 5 | 4 | 4 | 4 | 3 | 4 | 3 | 4 | 3 | 5 | 2 | 4 | 3 | 3.7 ± 1.2 |
|  | mean ± SD | 3.9  ± 1.1 | 3.3  ± 1.7 | 3.4  ± 1.3 | 3.8  ± 1.2 | 3.1  ± 1.6 | 3.1  ± 1.1 | 2.8  ± 1.2 | 3.6  ± 1.0 | 3.6  ± 1.0 | 3.4  ± 1.1 | 3.1  ± 1.5 | 3.0  ± 1.1 | 3.8  ± 1.2 | 2.8  ± 1.2 | 2.9  ± 1.2 | 3.4  ± 1.3 | 3.1  ± 1.4 | 3.1  ± 1.1 | 3.1 ± 0.9 |
| BH = breath-holding, FB= free-breathing, MOCO = Motion control, MOLLI = modified look-locker inversion-recovery, RV = right ventricular, SD = standard deviation  1= non-diagnostic / strongly pronounced, 2= poor / moderately pronounced, 3= fair / slightly pronounced, 4= good / hardly pronounced, 5= excellent / not present at all | | | | | | | | | | | | | | | | | | | | |

a)

| Rapid T1FLASH | | visibility of | | | | | | sharpness of RV wall | | | diagnostic  confidence | | | motion artefact | | | | | | mean ±  SD |
| --- | --- | --- | --- | --- | --- | --- | --- | --- | --- | --- | --- | --- | --- | --- | --- | --- | --- | --- | --- | --- |
|  |  | endocardial contour | | | epicardial contour | | |  |  |  |  |  |  | myocardial capping | | | loss of integrity | | |  |
|  | no. | basal | mid-ventricular | apical | basal | mid-ventricular | apical | basal | mid-ventricular | apical | basal | mid-ventricular | apical | basal | mid-ventricular | apical | basal | mid-ventricular | apical |  |
| BH | 1 | 5 | 4 | 3 | 5 | 5 | 4 | 5 | 5 | 5 | 5 | 4 | 4 | 5 | 5 | 3 | 5 | 5 | 4 | 4.3 ± 1.2 |
|  | 2 | 4 | 5 | 3 | 4 | 5 | 4 | 3 | 4 | 3 | 4 | 4 | 4 | 4 | 4 | 4 | 4 | 5 | 4 | 3.8 ± 1.1 |
|  | 3 | 4 | 5 | 4 | 4 | 5 | 3 | 4 | 4 | 4 | 4 | 5 | 4 | 5 | 5 | 5 | 4 | 5 | 5 | 4.2 ± 1.1 |
|  | 4 | 4 | 4 | 4 | 4 | 4 | 4 | 5 | 5 | 4 | 4 | 4 | 4 | 5 | 5 | 5 | 3 | 4 | 4 | 4.0 ± 1.1 |
|  | 5 | 4 | 4 | 4 | 4 | 4 | 4 | 3 | 3 | 4 | 3 | 3 | 3 | 5 | 5 | 5 | 4 | 4 | 4 | 3.7 ± 1.1 |
|  | 6 | 4 | 5 | 5 | 5 | 5 | 5 | 4 | 5 | 5 | 4 | 5 | 5 | 5 | 5 | 5 | 4 | 5 | 5 | 4.5 ± 1.1 |
|  | 7 | 4 | 5 | 5 | 4 | 5 | 5 | 5 | 5 | 5 | 4 | 5 | 5 | 4 | 5 | 5 | 4 | 5 | 5 | 4.5 ± 1.1 |
|  | 8 | 5 | 4 | 4 | 5 | 5 | 4 | 4 | 3 | 4 | 4 | 4 | 4 | 5 | 5 | 5 | 4 | 4 | 4 | 4.1 ± 1.1 |
|  | 9 | 5 | 5 | 4 | 5 | 5 | 4 | 5 | 5 | 5 | 5 | 5 | 4 | 4 | 5 | 5 | 5 | 5 | 3 | 4.4 ± 1.2 |
|  | 10 | 5 | 5 | 5 | 5 | 5 | 5 | 3 | 4 | 4 | 5 | 5 | 5 | 5 | 5 | 5 | 4 | 5 | 5 | 4.5 ± 1.2 |
|  | 11 | 4 | 4 | 4 | 4 | 4 | 4 | 4 | 3 | 4 | 4 | 4 | 4 | 5 | 5 | 4 | 4 | 3 | 4 | 3.8 ± 1.0 |
|  | 12 | 5 | 5 | 5 | 5 | 5 | 5 | 5 | 5 | 5 | 5 | 5 | 5 | 5 | 5 | 5 | 5 | 5 | 5 | 4.7 ± 1.1 |
|  | 13 | 5 | 5 | 5 | 5 | 5 | 4 | 3 | 4 | 4 | 5 | 5 | 4 | 5 | 5 | 5 | 5 | 5 | 4 | 4.4 ± 1.2 |
|  | 14 | 4 | 5 | 4 | 5 | 5 | 5 | 5 | 5 | 4 | 5 | 5 | 5 | 5 | 5 | 5 | 5 | 5 | 5 | 4.6 ± 1.1 |
|  | mean ± SD | 4.4  ± 0.5 | 4.6  ± 0.5 | 4.2  ± 0.7 | 4.6  ±0.5 | 4.8  ± 0.4 | 4.3  ± 0.6 | 4.1  ± 0.8 | 4.3  ± 0.8 | 4.3  ± 0.6 | 4.4  ± 0.6 | 4.5  ± 0.6 | 4.3  ± 0.6 | 4.8  ± 0.4 | 4.9  ± 0.3 | 4.6  ± 0.6 | 4.3  ± 0.6 | 4.6  ± 0.6 | 4.4  ± 0.6 | 4.2 ± 0.3 |
| FB | 1 | 4 | 4 | 3 | 5 | 3 | 3 | 4 | 4 | 4 | 4 | 4 | 3 | 3 | 3 | 2 | 4 | 3 | 3 | 3.5 ± 0.7 |
|  | 2 | 3 | 4 | 3 | 3 | 4 | 4 | 3 | 4 | 4 | 3 | 3 | 3 | 3 | 3 | 3 | 3 | 3 | 3 | 3.3 ± 0.4 |
|  | 3 | 2 | 3 | 4 | 1 | 3 | 4 | 2 | 3 | 3 | 1 | 3 | 3 | 4 | 1 | 3 | 1 | 3 | 3 | 2.6 ± 1.0 |
|  | 4 | 2 | 3 | 3 | 3 | 2 | 2 | 3 | 4 | 4 | 2 | 3 | 2 | 2 | 4 | 2 | 2 | 3 | 2 | 2.7 ± 0.7 |
|  | 5 | 1 | 2 | 2 | 1 | 2 | 2 | 2 | 2 | 2 | 1 | 1 | 1 | 1 | 1 | 1 | 1 | 1 | 1 | 1.4 ± 0.5 |
|  | 6 | 4 | 5 | 5 | 5 | 4 | 4 | 4 | 5 | 5 | 4 | 4 | 4 | 5 | 3 | 2 | 4 | 4 | 4 | 4.2 ± 0.7 |
|  | 7 | 2 | 3 | 1 | 2 | 3 | 1 | 2 | 3 | 3 | 2 | 3 | 1 | 2 | 3 | 1 | 2 | 3 | 1 | 2.1 ± 0.8 |
|  | 8 | 3 | 3 | 3 | 3 | 3 | 3 | 2 | 2 | 2 | 2 | 3 | 2 | 1 | 1 | 1 | 2 | 2 | 2 | 2.2 ± 0.7 |
|  | 9 | 1 | 2 | 3 | 1 | 2 | 3 | 4 | 3 | 3 | 1 | 1 | 2 | 1 | 2 | 3 | 1 | 1 | 2 | 2.0 ± 0.9 |
|  | 10 | 3 | 3 | 3 | 3 | 4 | 4 | 2 | 3 | 3 | 3 | 3 | 3 | 1 | 2 | 2 | 2 | 3 | 4 | 2.8 ± 0.7 |
|  | 11 | 1 | 1 | 3 | 1 | 1 | 3 | 2 | 3 | 2 | 1 | 1 | 3 | 1 | 1 | 2 | 1 | 1 | 2 | 1.7 ± 0.8 |
|  | 12 | 4 | 4 | 4 | 4 | 4 | 4 | 4 | 4 | 4 | 4 | 4 | 4 | 4 | 4 | 4 | 4 | 4 | 4 | 4.0 ± 0.0 |
|  | 13 | 4 | 4 | 3 | 5 | 4 | 3 | 3 | 3 | 3 | 5 | 4 | 3 | 2 | 3 | 3 | 4 | 3 | 3 | 3.4 ± 0.7 |
|  | 14 | 3 | 3 | 4 | 3 | 3 | 4 | 3 | 4 | 4 | 3 | 3 | 4 | 3 | 2 | 3 | 2 | 2 | 3 | 3.1 ± 0.6 |
|  | mean ± SD | 2.6  ± 1.1 | 3.1  ± 1.0 | 3.1  ± 0.9 | 2.9  ± 1.5 | 3.0  ± 0.9 | 3.1  ± 0.9 | 2.9  ± 0.8 | 3.4  ± 0.8 | 3.3  ± 0.9 | 2.6  ± 1.3 | 2.9  ± 1.1 | 2.7  ± 1.0 | 2.4  ± 1.3 | 2.4  ± 1.0 | 2.3  ± 0.9 | 2.4  ± 1.2 | 2.6  ± 1.0 | 2.6  ± 1.0 | 2.8 ± 0.8 |
| BH = breath-holding, FB= free-breathing, FLASH = fast low angle shot, RV = right ventricular, SD = standard deviation  1= non-diagnostic / strongly pronounced, 2= poor / moderately pronounced, 3= fair / slightly pronounced, 4= good / hardly pronounced, 5= excellent / not present at all | | | | | | | | | | | | | | | | | | | | |

b)
